# The Effect and Mechanism of POSTN and Its Alternative Splicing on the Apoptosis of Myocardial Cells in Acute Myocardial Infarction: A Study in Vitro

**DOI:** 10.1007/s12013-023-01157-w

**Published:** 2023-08-12

**Authors:** Xuemei Liu, Zulikaier Tuerxusssn, Yumaierjiang Balati, Pengfei Gong, Ze Zhang, Zhen Bao, Yuchun Yang, Pengyi He, Muhuyati

**Affiliations:** 1 Department of Respiratory Medicine, The First Affiliated Hospital of Xinjiang Medical University, Urumqi, 830054 China; 2 The Second Department of Coronary Heart Disease, the First Affiliated Hospital of Xinjiang Medical University, Urumqi, 830054 China; 3 Department of Integrated Cardiology, The First Affiliated Hospital of Xinjiang Medical University, Urumqi, 830054 China

**Keywords:** Acute myocardial infarction, POSTN, Alternative splicing, Apoptosis

## Abstract

Our study aimed to investigate key molecular targets in the pathogenesis of AMI, and provide new strategy for the treatment. In this work, the myocardial ischemia and hypoxia model was constructed by using HL-1 mouse cardiomyocytes. The over-expressing POSTN wild-type, mutant and negative control lentiviruses (GV492-POSTNWT,GV492-POSTN-MUT, GV492-NC) was conducted and transfected. Cardiomyocytes were examined for cell proliferation and apoptosis to explore the effects of POSTN and its alternative splicing. The endoplasmic reticulum stess-related apoptosis proteins were selected and detected. We found that POSTN could promote the proliferation of normal and hypoxic cardiomyocytes and inhibit their apoptosis. The mechanism by which POSTN inhibited cardiomyocyte apoptosis may be through inhibiting the GRP78-eIF2α-ATF4-CHOP pathway of endoplasmic reticulum stress. Alternative splicing of POSTN could inhibit the apoptosis of ischemic and hypoxic cardiomyocytes, and its mechanism needs to be confirmed by further studies. We drawed the conclusion that POSTN might be a potential therapeutic target for AMI.

## Introduction

Acute myocardial infarction (AMI) is a cardiovascular emergency in which the myocardium is irreversibly damaged. Its pathogenesis is mainly due to coronary atherosclerotic stenosis, plaque rupture, platelet aggregation and thrombus formation, leading to interruption of coronary blood flow and local myocardial ischemia, hypoxia and ultimately myocardial cell death. Its characteristics of acute onset, rapid progression, high morbidity and mortality severely threaten the health of human beings. At present, the treatment of acute myocardial infarction mainly relies on early surgical interventions to restore coronary blood flow as early as possible, in order to reduce myocardial cell death and fibrosis, thereby to improve the prognosis of heart failure after myocardial infarction. However, effective drug treatment is still lacking. Therefore, it is particularly meaningful to search for important molecular targets in the pathogenesis of AMI in order to provide new strategies for the treatment.

Periostin, encoded by the POSTN gene, is a secreted protein that exists in the extracellular matrix and is highly expressed in osteoblasts and their precursor cells [1, 2]. Current studies have found that POSTN is widely expressed in many human tissues including the heart, liver, kidney, adrenal gland, thyroid and so on [3]. In addition, periostin has been found to be associated with various diseases, such as tumors, inflammatory, respiratory and cardiovascular diseases [4, 5].

In our previous study, we found significant differences on the expression of POSTN and its alternative splicing (AS) between AMI and sham-operated mice groups via high-throughput RNA sequencing [6]. In order to verify the potential role of POSTN and its alternative splicing in AMI, we examined the plasma POSTN protein levels of AMI patients at different stages, and also the expression level of POSTN gene in peripheral blood mononuclear cell (PBMC) within 24 h of myocardial infarction [7]. The results showed that POSTN protein level was significantly decreased in the early stage of AMI (within 24 h), then started to increase for the next 7 days, and reached its normal level in about 30 days. Moreover, the expression of POSTN in PBMC of AMI patients was significantly decreased within the first 24 h, indicating that POSTN was closely related to the pathogenesis of AMI, however the mechanism was unclear. Previous studies have showed that POSTN could promote the proliferation of cardiomyocytes and the formation of myocardial fibrosis after AMI. Another study reported that POSTN could inhibit the apoptosis of melanoma osteoblasts by inhibiting endoplasmic reticulum stress [8]. Cardiomyocyte apoptosis is one of the important molecular phenotypes and outcomes after AMI, but whether or not POSTN is related to cardiomyocyte apoptosis is still unclear. In this study, the effect and mechanism of POSTN and its alternative splicing on cardiomyocyte apoptosis were studied to further explore the role of POSTN and its alternative splicing in the pathogenesis of AMI.

## Materials and Methods

### Cell Culture and Treatment

The HL-1 mouse cardiomyocyte cells were obtained from Yagi Biology Company, which were cultured in Dulbecco’s modified Eagle’s medium (DMEM) with 10% fetal bovine serum (FBS) and 1% penicillin/streptomycin (P/S) at 37 °C, 5% CO_2_ with saturated humidity. We continued to culture the HL-1 mouse cardiomyocyte cells in complete cell culture media, selected cells in good growth and made single cell suspension with 5 × 10^4^ cells/ml. We then used CCK-8 cell proliferation/toxicity testing kit (FC101-03, TransGen Biotech, Beijing, China) to assess the growth after 1 week. Finally, we measured the optical density (OD) value at 450 nm to draw out the cell growth curve, using microplate reader (xMarkTM, Bio-Rad, the USA).

### Construction of Ischemic Hypoxic Cardiomyocyte Model

#### Hypoxia treatment

The cultured cells were transferred to serum-free media, and then further cultured at 37 °C in gas containing 1% O_2_, 4% CO_2,_ and 95% N_2_ for 3, 6, 12, 24 h respectively. The control cells were cultured at 37 °C in normal condition for the same time period.

#### Cell proliferation assay

After the hypoxia treatment, the culture media were discarded, then 100 μl of 10% CCK-8 solution was added to each well, and continued culture for 1 h. Lastly the OD value was measured at 450 nm to assess cell growth curve.

#### Lactate dehydrogenase (LDH) assay

The LDH detection kits (Nanjing Jiancheng, A020-2-2) were used for LDH detection on the treated culture supernatant. The formula for LDH calculation is: LDH (U/L) = (measured OD value - control OD value)/(standard OD - blank OD value) × standard concentration (0.2 μmol/L) × 1000.

### Construction of the Overexpression Lentiviral Vectors

Primers were designed using the sequence of POSTN and the alternative splicing of POSTN in GenBank, which contained the BamHI and AgeI restriction enzymes sites. The primers are shown in Table 1a, b. RT-PCR was used to clone the gene of POSTN and POSTN alternative splicing. The RT-PCR products were resolved by 1% agarose gel electrophoresis, and then purified, sequenced and cloned into the lentiviral vector, GV492, to produce the POSTN (GV492-POSTN-WT) and POSTN alternative splicing (GV492-POSTN-MUT) overexpression lentiviral vector. An empty vector was used as the negative control (GV492-NC). The construction of vector lentiviruses was conducted by Genechem Co., Ltd (Shanghai, China).

**Table 1:** a. Primer of POSTN gene. b Primer of POSTN-AS gene

Primer	Sequence (5’-3’)
**a**	
POSTN-F	AGGTCGACTCTAGAGGATCCCGCCACCATGGTTCCTCTCCTGCCCTTATATGC
POSTN-R	TCCTTGTAGTCCATACCCTGAGAACGGCCTTCTCTTGATCG
**b**	
POSTN-AS-F	AGGTCGACTCTAGAGGATCCCGCCACCATGGTTCCTCTCCTGCCCTTATATGCTC
POSTN-AS-R	TCCTTGTAGTCCATACCCTGAGAACGGCCTTCTCTTGATCGTCTTC

### Exploration on the Optimal Condition of Cell Transfection

MOI (multiplicity of infection) refers to the ratio of the number of viruses to the number of cells during infection. The formula to calculate MOI is: MOI=viral titer (TU/ml) × viral volume (ml)/cell number.

We selected three potential MOI value, 1, 10, 100 after literature review, and calculated the viral titers, 1 × 10^6^ TU/ml, 1 × 10^7^ TU/ml, 1 × 10^8^ TU/ml, respectively, using the formula above (viral volume was 10 μl). We also compared the transfection efficiency using two different transfection reagents, HitransG A (Reagent A) and HitransG P (Reagent P) (Genechem, Shanghai, China).

We titrated the GV492-NC culture to 1 × 10^6^ TU/ml, 1 × 10^7^ TU/ml, 1 × 10^8^ TU/ml for 50 μl each using serum-free media, then mixed the viruses and reagents as shown in Table 2. After 12 h of transfection the viruses were transferred back to normal culture media. We determined the optimal transfection condition by observing the fluorescence intensity under inverted fluorescence microscope (Eclipse TS100-F, Nikon, Japan). The optimal condition should have high fluorescence intensity with a transfection efficiency close to 80% and good cell growth. The corresponding MOI to such condition was then chosen for subsequent procedures.

**Table 2:** Testing groups and transfection conditions

Transfection condition	Group M	Group A	Group P
MOI = 1	Normal media: 90 μl	Normal media: 86 μl	Normal media: 86 μl
1 × 10^6^ TU/ml			
	Virus: 10 μl	Virus: 10 μl	Virus: 10 μl
		Reagent A: 4 μl	Reagent P: 4 μl
MOI = 10	Normal media: 90 μl	Normal media: 86 μl	Normal media: 86 μl
1 × 10^7^ TU/ml			
	Virus: 10 μl	Virus: 10 μl	Virus: 10 μl
		Reagent A: 4 μl	Reagent P: 4 μl
MOI = 100	Normal media: 90 μl	Normal media: 86 μl	Normal media: 86 μl
1 × 10^8^ TU/ml			
	Virus: 10 μl	Virus: 10 μl	Virus: 10 μl
		Reagent A: 4 μl	Reagent P: 4 μl

### Cell Transfection and Quantitative Real-time PCR (qRT-PCR)

We transfected the cultured cardiomyocytes with the GV492-POSTN-WT and GV492-POSTN-MUT using the optimal condition. After discarding the culture media, the cells were digested using 1 ml of TRIzol™ Reagent (15596026, Ambion, the USA). Then the RNA was extracted using TRIzol protocol for each group, and 5X All-In-One RT MasterMix (with AccuRT Genomic DNA Removal Kit) Reverse Transcription Kit (G492, abm, the USA) was utilized to convert RNA to cDNA. The POSTN gene in cDNA was amplified using SYBR Green Real-time kit (TaKaRa, Dalian, China) on an ABI7500 Fast RT-PCR system (ABI, the USA) according to the manufacturer’s instructions. Mouse β-actin was used as the endogenous control. The primers are shown in Table 3a, b. The relative gene expression was calculated by the 2^-△△Ct^ method. The POSTN alternative spicing gene in cDNA was amplified using 2×EasyTaq PCR SuperMix (+dye) (AS111, TransGen Biotech, Beijing, China) on a MyCycler Thermal Cycler PCR system (Bio-Rad, the USA) according to the manufacturer’s instructions. The PCR products were checked by electrophoresis with 2% agarose gel and observed by ethidium bromide (EtBr) staining. Finally the electrophoretic bands were scanned and the grayscale values were obtained using Adobe Photoshop to semi-quantify the expression levels of the POSTN alternative splicing.

**Table 3:** a Primers of POSTN and β-actin. b Primer of POSTN-AS

Primer	Sequence (5'-3')	Product (bp)
**a**		
POSTN-F	GGACCTTGTTTGCACCAACC	147
POSTN-R	CGGGTTCGAATCCCTTTCCA	
Mouse β-actin-F	GGCTGTATTCCCCTCCATCG	154
Mouse β-actin-R	CCAGTTGGTAACAATGCCATGT	
**b**		
POSTN-AS-F	ACAAACTCCTCTATCCAGC	292/211
POSTN-AS-R	TCTGTCACCGTTTCGCCTTC	

### The Effects of POSTN and its Alternative Splicing on Cardiomyocytes

#### Cell proliferation assay

We utilized CCK-8 cell proliferation assay to measure the OD values of normal and ischemic hypoxic cardiomyocytes transfected with GV492-POSTN-WT, GV492-POSTN-MUT. The method was as stated in previous sections.

#### Cell apoptosis assay

We used Annexin V-PE/7-AAD Cell Apoptosis Testing Kit (559763, BD, the USA) to measure the apoptosis of the cardiomyocytes. Transfected cells in each group were transferred into 15 ml centrifuge tubes, washed twice with Phosphate Buffer Saline (PBS) solution, and then trypsinized (0.25% Trypsin-EDTA, 25200-056, GIBCO, the USA). Then we added 500 μl of 1 × Binding Buffer to the centrifuge tube to resuspend the cells, and made them into single-cell suspension through grit 200 filter. We then added 5 μl of Annexin V-PE and 10 μl of 7-AAD into each tube and mixed gently. After incubating for 15 min at 4 °C in the dark, the samples were analyzed by flow cytometry (LSRFortessa, BD, the USA).

#### Western blot

Cardiomyocytes in each group were collected and total proteins were extracted using RIPA RIPA Lysis and Extraction Buffer (AR0105, Boster Biological Technology, Wuhan, China), and the protein concentration was determined using Easy II Protein Quantitative BCA kit (DQ111-01, TransGen Biotech, Beijing, China). The OD values were measured at 562 nm, and the concentration of proteins were calculated with the standard curve. The protein samples were pre-processed using 5×SDS-PAGE loading buffer (with β-Mercaptoethanol) (C05-03001, Bioss, Beijing, China), isolated with electrophoresis (10% gel for eIF2α and GRP78; 12% gel for ATF4 and β-actin; 15% gel for CHOP, BCL-2 and BAX), and transferred to ploy membrane (0.45 um polyvinylidene fluoride PVDF membrane [IPVH00010, Millipore, the USA] for eIF2α, ATF4, GRP78 and β-actin; 0.22 um PVDF membrane [ISEQ00010, Millipore, the USA] for CHOP, BCL-2 and BAX). The membrane was blocked using blocking solution containing 5% skim milk for 1 h, and incubated with primary and secondary antibodies (Table S1). Mouse β-actin was used as the internal reference for other proteins. The protein bands were visualized using chemiluminescence kit (Chemiscope 3000, Clinx Science Instruments, Shanghai, China) to assess the expression levels of eIF2α, ATF4, CHOP, GRP78, BCL-2 and BAX in each group.

### Statistical Analysis

We used SPSS 20.0 to conduct the statistical analysis on the level of each gene in each group, with measurement data using mean ± standard deviation (χ ®±s) and enumeration data using proportion. The measurement data were tested for normality and proved to be normally distributed. Therefore two-tailed independent samples *t*-test was used with significance level set at *p* < 0.05.

## Results

### The HL-1 Cardiomyocytes Grew Logarithmically

**Fig. 1:**
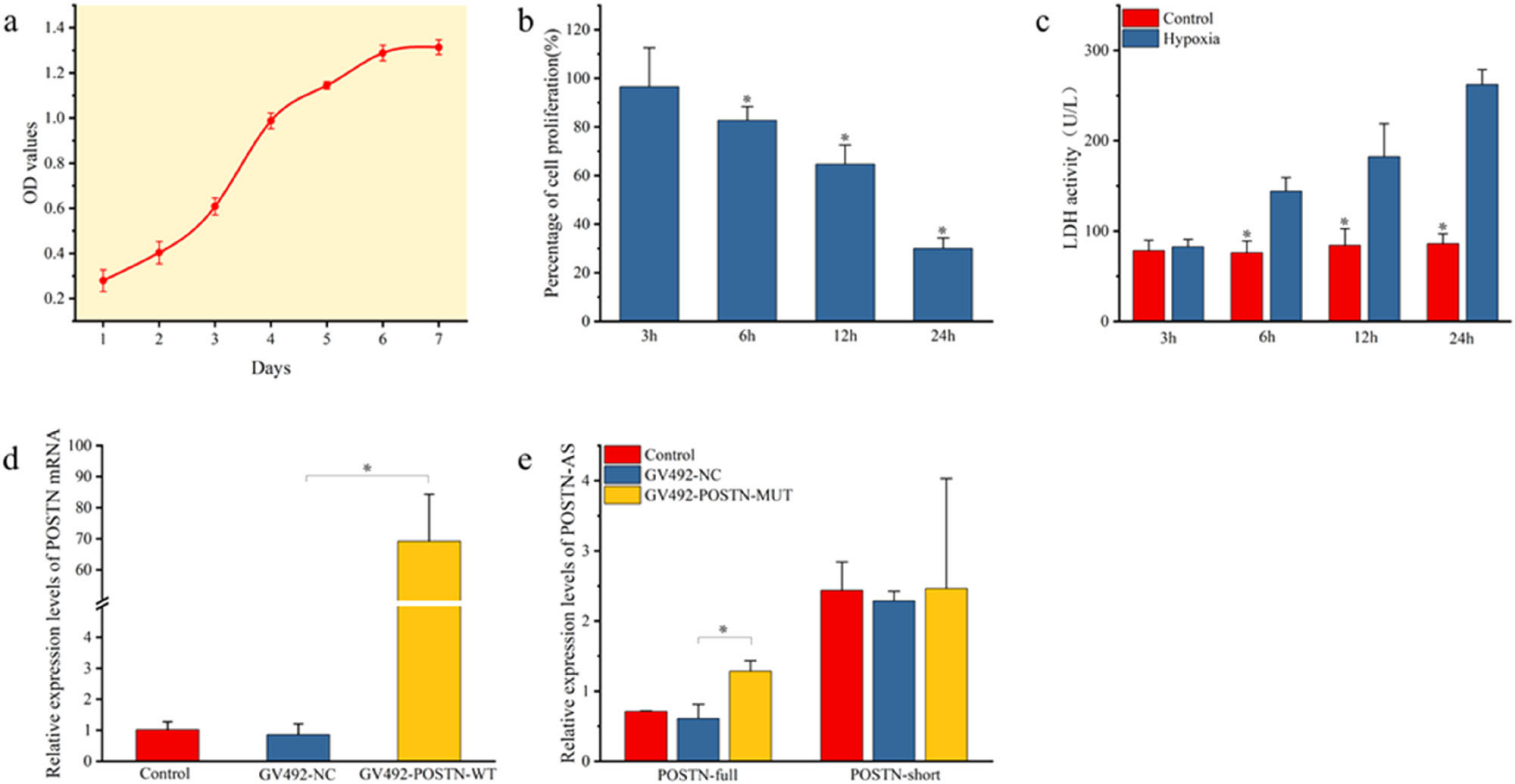
Growth of the cardiomyocytes, effects of different hypoxia treatment and expression levels of the POSTN and POSTN-AS. **a** The growth curve of HL-1 cells. **b** The cell proliferation of HL-1 cardiomyocytes at different hypoxia treatment time, and the percentage of cell proliferation of the control at each time was 100%. **c** The effects of different hypoxia treatment time on LDH activity of HL-1 cardiomyocytes. **d** POSTN expression level in cardiomyocytes transfected with GV492-POSTN-WT by qRT-PCR. **e** POSTN alternative splicing expression level in cardiomyocytes transfected with GV492-POSTN-MUT shown as gray value of bands by PCR; POSTN-full (292 bp) is POSTN alternative splicing studied in this paper; POSTN-short (211 bp) is the reference transcript. *Represents *p* < 0.05. GV492-NC is the negative control lentivirus; GV492-POSTN-WT is POSTN wild-type (POSTN) overexpressed lentivirus; GV492-POSTN-MUT is POSTN mutant-type (POSTN alternative splicing) overexpressed lentivirus

The growth curve was drawn according to the OD values (Table S2) of HL-1 cardiomyocytes after culture for 7 days (Fig. 1a). the cells grew well with logarithmic growth and were used for subsequent studies.

### The Optimal Hypoxia Treatment Time for Cardiomyocyte Hypoxia Model

The hypoxia treatment of cells for 3 h had little effect on cell proliferation and LDH. At 6, 12 and 24 h of hypoxia treatment, cell proliferation was significantly inhibited and the LDH level was significantly increased (Tables S3, 4, Fig. 1b, c). The results suggested that hypoxia treatment for more than 6 h significantly inhibited the proliferation of cardiomyocytes and caused obvious oxidative stress damage. At 24 h, the inhibitory effect of hypoxia on cell proliferation was strongest, however, the OD value of cells was close to 1/4 of the control, indicating that the survival rate was too low to yield enough cells for subsequent studies. Therefore, 24 h was not the optimal hypoxia treatment time. Between 6 and 12 h of hypoxia treatment, the LDH of cells increased significantly at 6 h, therefore the optimal hypoxia treatment time of cardiomyocytes in the model of ischemia and hypoxia was 12 h.

### The Construction of Lentiviral Vector and Transfection

POSTN was highly expressed in cardiomyocytes that transfected with GV492-POSTN-WT (Table S5, Fig. 1d). The POSTN alternative splicing (POSTN-full) was also highly expressed in cardiomyocytes that transfected with GV492-POSTN-MUT (Table S6, Fig. 1e). The results showed that both GV492-POSTN-WT and GV492-POSTN-MUT were successfully constructed and the cardiomyocytes were also successfully transfected.

### The Best Transfection Conditions of Lentivirus

**Fig. 2:**
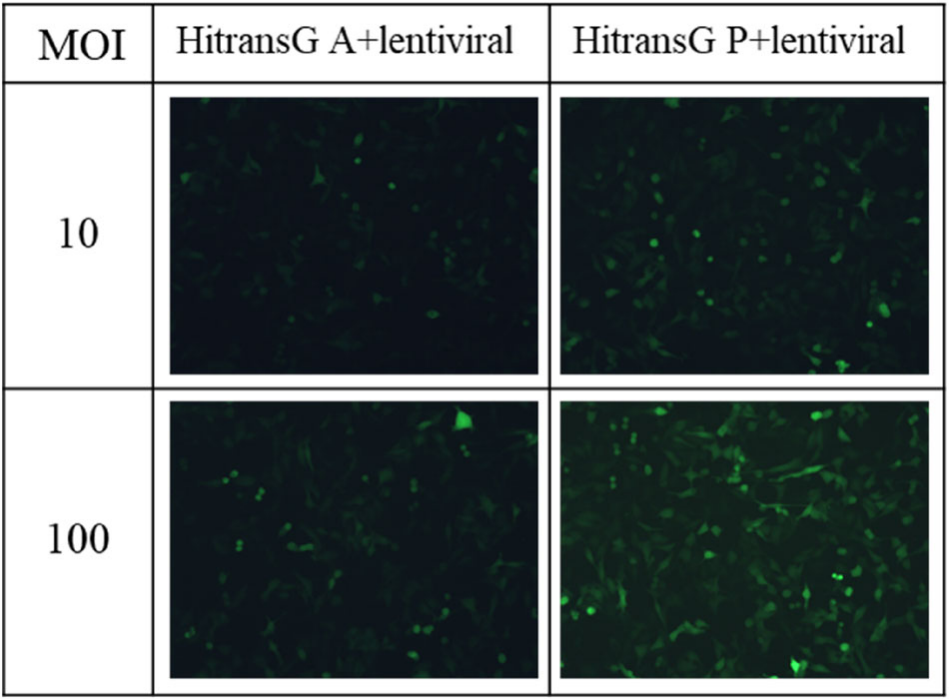
Transfection effects at different MOIs with different reagents

According to the influence of different MOI values and the addition of different transfection reagents on cell growth, it was found that at MOI = 100 with Reagent P, the lentiviruses had the highest infection efficiency on HL-1 cells with the best cell growth. Therefore, this condition was determined as the optimal infection condition (Fig. 2).

### POSTN Promoted the Proliferation of Normal and Hypoxic Cardiomyocytes

**Fig. 3:**
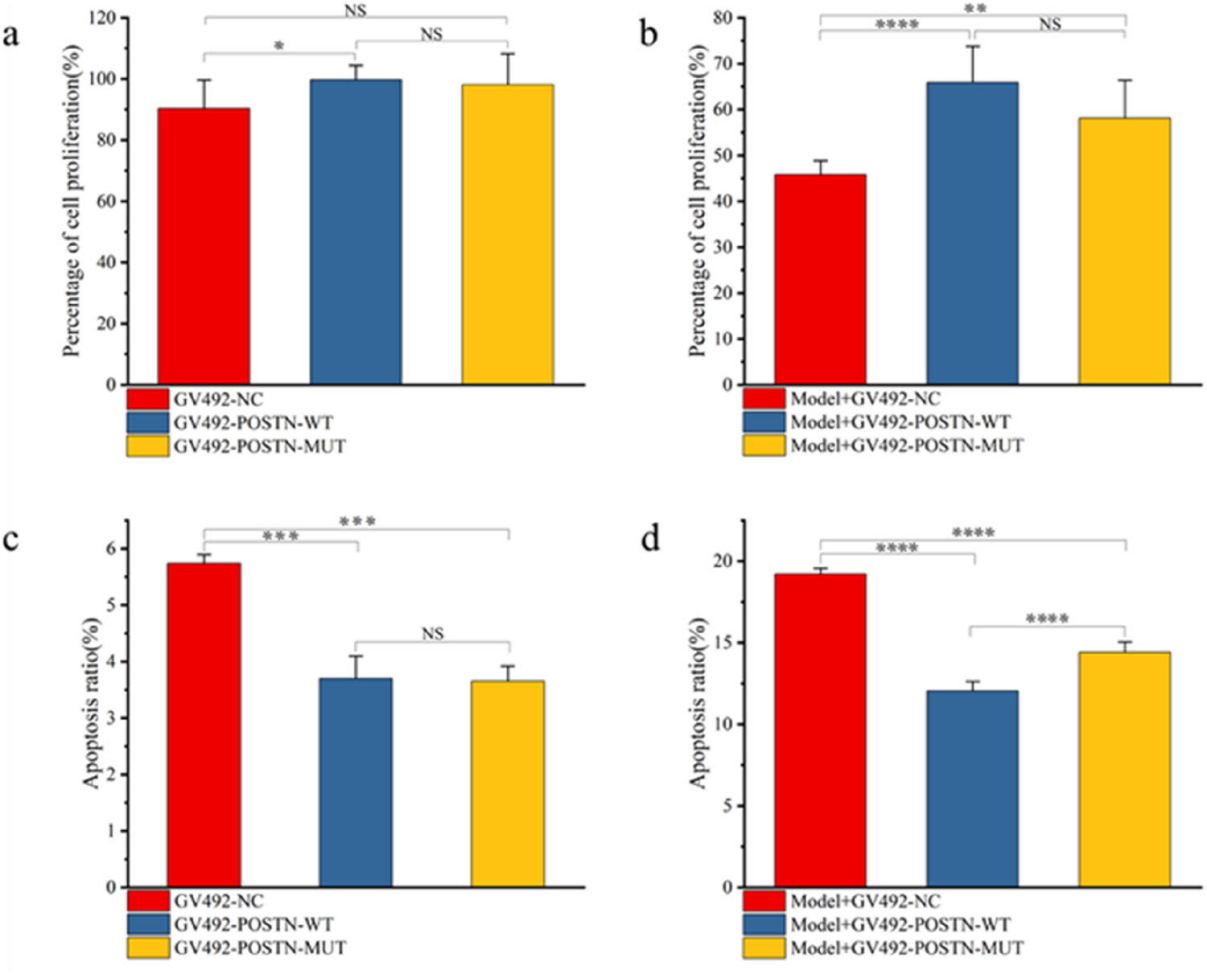
Effects of GV492-POSTN-WT and GV492-POSTN-MUT on the proliferation and apoptosis of cardiomyocytes. **a** The proliferation of normal cardiomyocytes transfected with GV492-POSTN-WT and GV492-POSTN-MUT. **b** The proliferation of ischemic hypoxic cardiomyocytes transfected with GV492-POSTN-WT and GV492-POSTN-MUT. **c** Effect of GV492-POSTN-WT and GV492-POSTN-MUT on the apoptosis of normal cardiomyocytes. **d** Effect of GV492-POSTN-WT and GV492-POSTN-MUT on the apoptosis of hypoxic cardiomyocytes. *Represents *p* < 0.05, **represents *p* < 0.01, ***represents *p* < 0.001, ****represents *p* < 0.0001, NS represents no statistical difference, and the “model” is a shorthand for “ischemic hypoxic myocardial cell model”

The proliferation of normal cardiomyocytes transfected with GV492-POSTN-WT was significantly up-regulated (*p* < 0.05), while the proliferation of those transfected with GV492-POSTN-MUT was not significantly changed. In addition, the proliferation of normal cardiomyocytes transfected with GV492-POSTN-WT and GV492-POSTN-MUT did not show significant difference between the two groups, indicating that only the POSTN could promote the proliferation of normal cardiomyocytes (Table S7, Fig. 3a). Likewise, the proliferation of ischemic hypoxic cardiomyocytes transfected with GV492-POSTN-WT (*p* < 0.0001) or GV492-POSTN-MUT (*p* < 0.01) was significantly up-regulated, but there was also no significant difference between the two groups. As the alternative splicing of POSTN studied in this paper occurs in a cassette exon, which is a segment added to the original POSTN gene, only a significant difference between these two groups would suggest that the observed effect was caused by the fragment added to the original POSTN gene, in other words, the POSTN alternative splicing would have additional effect apart from that caused by the original POSTN gene. Therefore, the results indicated that only the POSTN could promote the proliferation of normal or ischemic hypoxic cardiomyocytes, but not its alternative splicing. (Table S7, Fig. 3b).

### POSTN and its Alternative Splicing inhibited Cardiomyocytes Apoptosis

**Fig. 4:**
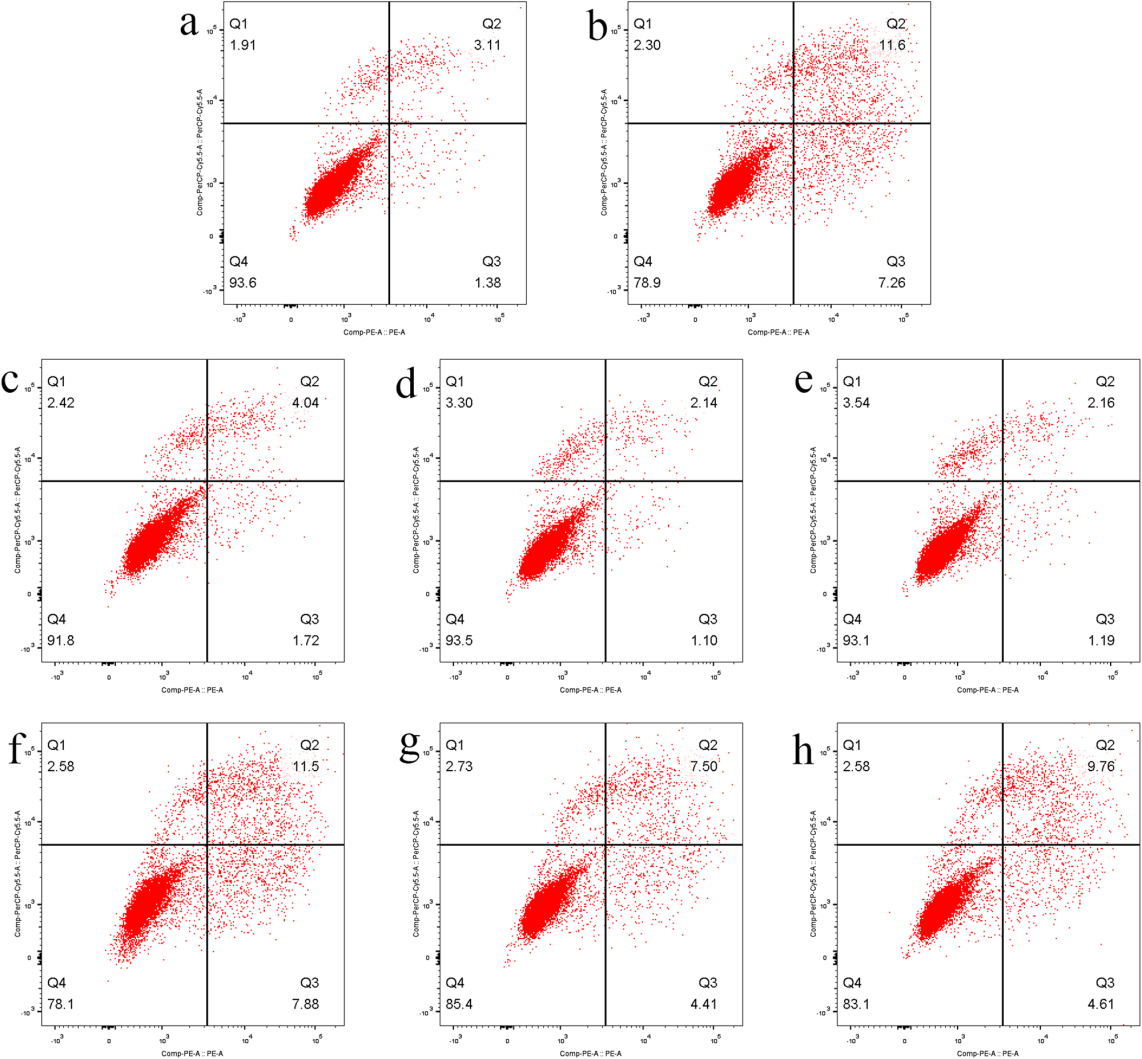
Cell apoptosis of cardiomyocytes under different conditions. The apoptosis of cardiomyocytes in (**a**) blank group, (**b**) ischemic hypoxic model group, (**c**) GV492-NC group, (**d**) GV492-POSTN-WT group, (**e**) GV492-POSTN-MUT group, (**f**) ischemic hypoxic model+GV492-NC group, (**g**) ischemic hypoxic model+GV492-POSTN-WT group and (**h**) ischemic hypoxic model+GV492-POSTN-MUT group. Note: x-axis represents Annexin V-PE, y-axis represents 7-AAD; Q1 represents cells with mechanical injury, Q2 represents cells in late apoptosis, Q3 represents cells in early apoptosis, Q4 represents cells without apoptosis (normal cells)

The apoptosis rates of normal cardiomyocytes transfected with GV492-POSTN-WT and GV492-POSTN-MUT were significantly decreased (*p* < 0.001), but there was no significant different between the two groups (Table S8, Fig. 3c). This indicated that the POSTN inhibited the apoptosis of normal cardiomyocytes, but not its alternative splicing. The apoptosis rates of ischemic hypoxic cardiomyocytes transfected with GV492-POSTN-WT and GV492-POSTN-MUT were significantly reduced (*p* < 0.0001), and the difference between the two groups was also statistically significant (*p* < 0.0001). The apoptosis rate of ischemic hypoxic cardiomyocytes transfected with GV492-POSTN-WT was lower (Table S8, Fig. 3d, Fig. 4). The results suggested that both POSTN and its alternative splicing could inhibit the apoptosis of ischemic hypoxic cardiomyocytes, and the inhibitory effect of POSTN was stronger.

### POSTN inhibited Cardiomyocytes Apoptosis through GRP78-eIF2α-ATF4-CHOP Pathway

Compared with normal cardiomyocytes transfected with GV492-NC, the expressions of elF2α, CHOP, GRP78 and BCL-2 in cardiomyocytes transfected with either GV492-POSTN-WT or GV492-POSTN-MUT were significantly different. The expressions of elF2α, CHOP and GRP78 were significantly decreased and BCL-2 was significantly increased in both GV492-POSTN-WT and GV492-POSTN-MUT transfected groups. The expressions of ATF4 and BAX were significantly decreased in GV492-POSTN-WT transfected group, however, in GV492-POSTN-MUT transfected group the expression tended to be lower but no significant difference was shown. There was no significant difference in the expression of the six proteins between the GV492-POSTN-WT and GV492-POSTN-MUT transfected groups. But in GV492-POSTN-MUT transfected group, elF2α, ATF4, CHOP, GRP78 and BAX showed a tendency of low expression, while BCL-2 showed a tendency of high expression, although without statistical significance.

**Fig. 5:**
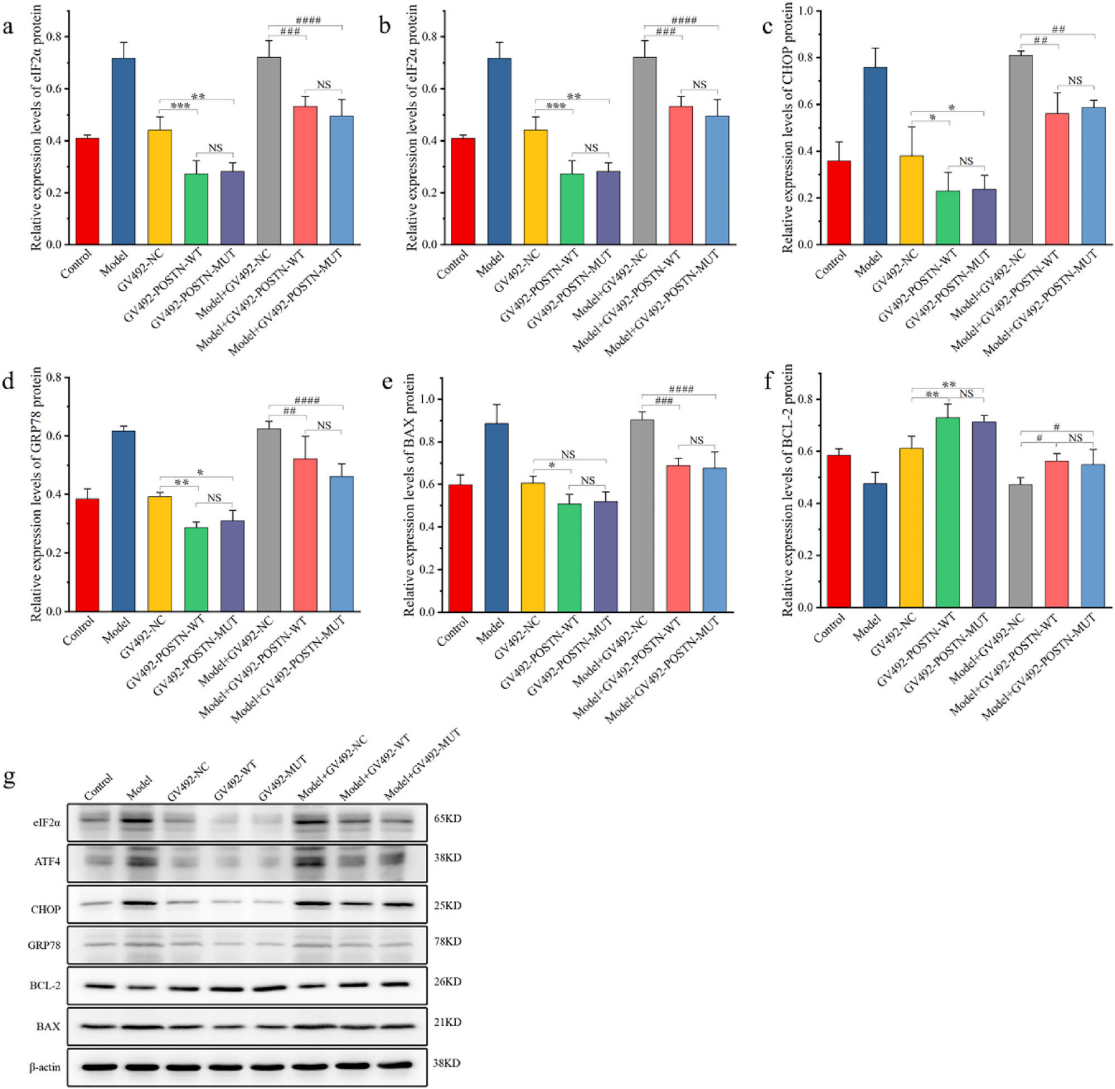
Expression levels of apoptosis-related proteins. **a**–**f** The relative expression levels of eIF2α, ATF4, CHOP, GRP78, BAX, BCL-2; * is compare with GV492-NC; # is compare with Model+GV492-NC; * represents *p* < 0.05; ** represents *p* < 0.01; ## represents *p* < 0.01; #### represents *p* < 0.0001; NS represents no statistically significant difference; (**g**) shows the WB electrophoresis of each protein

Similarly, there were significant differences in the expression levels of eIF2α, ATF4, CHOP, GRP78, BCL-2, and BAX between the ischemic hypoxic cells transfected with GV492-NC and those transfected with either GV492-POSTN-WT or GV492-POSTN-MUT. EIF2α, ATF4, CHOP, GRP78 and BAX were significantly decreased and BCL-2 was significantly increased, but there was no significant difference between the GV492-POSTN-WT and GV492-POSTN-MUT groups. (Tables S9, 10 and Fig. 5, Fig. S1).

## Discussion

Previous studies had found that POSTN was basically not expressed in the normal adult heart tissues, and highly expressed after cardiac injury such as myocardial infarction, pressure overload, and dilated cardiomyopathy [9, 10]. POSTN was also found to lowly expressed in the early stage of AMI patients’ plasma [11]. The role of POSTN in myocardial tissue and cells after AMI is still controversial [12–14].

Some researchers proposed POSTN could promote the proliferation of myocardial cells after infarction [15–17]. Another study showed that compared with the control group, knockdown of POSTN in AMI mice models significantly increased fibrosis and cardiomyocyte regeneration after myocardial infarction [18]. Nevertheless, other research found no obvious effect of POSTN on myocardial regeneration through in vivo and in vitro experiments [19], indicated that the ability of POSTN to induce cardiomyocyte proliferation remains controversial.

In our preceding study, RNA sequencing of myocardial tissue of AMI and Sham mice found that POSTN was lowly expressed in the myocardial tissue of AMI mice, while its alternative splicing was highly expressed, and the two expression trends were opposite. So does POSTN play opposite roles to its alternatively spliced transcripts in the mechanism of AMI? Through this study, we concluded that the effects of POSTN and alternatively spliced transcripts on normal and ischemic hypoxic cardiomyocytes were showing consistent trends. And in this part of the study, POSTN was lowly expressed in ischemic/ hypoxic cardiomyocytes, and POSTN alternative splicing was highly expressed in ischemic/ hypoxic cardiomyocytes, which was consistent with our previous detection results in animals.

Is this study we found that POSTN inhibited apoptosis, which was firstly reported in neoplastic diseases. Previous studies have shown that POSTN can inhibit the apoptosis of colon, liver, gastric, and pancreatic cancer cells, and promote tumor cell growth and metastasis [20–22]. Baril et al. found in their research that POSTN could promote the invasiveness of pancreatic cancer cells, and the effects were more obvious in hypoxic condition [23]. Liu et al. also found that down-regulating POSTN in hypoxic condition could facilitate liver cancer cell apoptosis [24]. Therefore, we could speculate that POSTN has anti-apoptosis effect on cancer cells, moreover, this effect is closely related to hypoxia. AMI features cardiomyocyte ischemia, hypoxia, and apoptosis, however, very few studies have been done to investigate the association between POSTN and apoptosis in AMI.

Endoplasmic reticulum stress (ERS)-mediated apoptosis has become a research hotspot in recent years [25–27]. One study showed that ERS played an important role in inducing cardiomyocyte apoptosis in a pressure overload mouse model of heart failure [28]. Bi X et al. suggested that ERS was involved in cardiomyocyte apoptosis by elucidating the role of the master regulator GRP78 in the unfolded protein response (URP) in myocardial ischemia/reperfusion injury [29]. Meng X et al. have confirmed that POSTN could protect osteoblasts by inhibiting the ERS-related eIF2α-ATF4 pathway and reducing the induction of melatonin on human osteoblast apoptosis [8]. ERS mainly induces apoptosis through three URP signal pathways, namely PERK pathway, IRE1 pathway and ATF6 pathway, and PERK-eIF2α-ATF4-CHOP is one of the PERK pathways [30, 31]. GRP78 is the major resident protein of the endoplasmic reticulum and is often used as an important marker for evaluating the function of the endoplasmic reticulum [32]. CHOP, a homologous protein of the pro-apoptotic transcription factor C/EBP, is a typical marker of endoplasmic reticulum stress and a downstream target of the UPR pathway [33, 34]. During ERS, due to increased phosphorylation of eIF2α, only specific mRNAs such as ATF4 and CHOP mRNA are translated, and ATF4 directly binds to the CHOP promoter and activates its transcription [28, 35, 36]. Therefore, we hypothesized that POSTN might affect cardiomyocyte apoptosis by regulating the eIF2α-ATF4-CHOP pathway of ERS.

In this study, the effect of POSTN and its alternative splicing on proliferation and apoptosis of normal and ischemic hypoxic cardiomyocytes were observed. POSTN promoted the proliferation of normal and hypoxic cardiomyocytes. POSTN alternative splicing showed a tendency of promoting the proliferation of normal and ischemic hypoxic cardiomyocytes compared with the control group, but there was no statistical significance. The effect of POSTN alternative splicing on cardiomyocytes needs further study to confirm. Apoptosis studies showed that POSTN could inhibit the apoptosis of normal and ischemic hypoxic cardiomyocytes, while POSTN splicing had no significant inhibitory effect on the apoptosis of normal cardiomyocytes, but had an inhibitory effect on the apoptosis of ischemic hypoxic cardiomyocytes. Therefore, we concluded that POSTN could promote the proliferation of AMI cardiomyocytes and inhibit the apoptosis of AMI cardiomyocytes. We simulated AMI cardiomyocytes through ischemic hypoxic cardiomyocyte modelling, and demonstrated that POSTN could promote the proliferation and inhibit the apoptosis of AMI cardiomyocytes, whilst the alternative splicing could inhibit the apoptosis of AMI cardiomyocytes. The promoting trend of POSTN alternative splicing on the proliferation of AMI cardiomyocytes was observed, but there was no statistical significance. However it would be premature to deny the effect of POSTN alternative splicing on AMI cardiomyocytes proliferation, instead, it needs to be confirmed by further experiments.

To explore the mechanism of POSTN and its alternative splicing inhibiting cardiomyocyte apoptosis, we examined ERS related proteins GRP78, eIF2α, ATF4, CHOP and apoptosis-related proteins BCL-2 and BAX, and found that POSTN inhibited the expression of GRP78, eIF2α, ATF4, CHOP proteins, while the expression of anti-apoptotic protein BCL-2 was up-regulated and the expression of pro-apoptotic protein BAX was down-regulated. These results indicated that POSTN inhibited cardiomyocyte apoptosis by inhibiting GRP78-eIF2α-ATF4-CHOP pathway. The same trend with POSTN alternative splicing was observed in both normal and ischemic hypoxic cardiomyocytes, but the role of POSTN alternative splicing could not be proved because there was no statistical difference between POSTN and POSTN alternative splicing. However, it is also premature to deny the possible influence of POSTN alternative splicing on this pathway, which calls for further study to confirm. It was concluded that POSTN inhibited cardiomyocyte apoptosis post AMI by inhibiting GRP78-eIF2α-ATF4-CHOP pathway, and POSTN had a protective effect on AMI cardiomyocytes.

In this study, we demonstrated that POSTN played a crucial role in the pathogenesis of AMI. POSTN could promote the proliferation of AMI cardiomyocytes, inhibit the apoptosis of AMI cardiomyocytes, suggesting a protective effect on AMI. We suspected that up-regulation of POSTN might play a role in the clinical treatment of AMI, which makes POSTN a potential biomarker and therapeutic target for AMI.

### Limitations

The limitation of our study was that we did not verify the effect of POSTN knockout or knockdown on cardiomyocytes proliferation and apoptosis, and there was no further in vivo confirmation. Through this study, we had some preliminary conclusions, and we will conduct a series of further studies to thoroughly verify the role of POSTN in the pathogenesis of AMI. We hope our research can lead to a new breakthrough in the diagnosis and treatment of AMI.

## Supplementary information

Supplementary information

## Data Availability

The datasets used or analyzed during the current study are available from the corresponding author on reasonable request.
