# Supplementary material for: The Effect and Mechanism of POSTN and Its Alternative Splicing on the Apoptosis of Myocardial Cells in Acute Myocardial Infarction: A Study in Vitro
Source: Cell Biochem Biophys. 2023 Aug 12;81(3):481–91. doi: 10.1007/s12013-023-01157-w (PMC10465634; doi:10.1007/s12013-023-01157-w)
Supplement: Supplementary file 1 — Supplementary information [file 12013_2023_1157_MOESM1_ESM.docx]

**Supplementary information**

**The effect and mechanism of POSTN and its alternative splicing on the apoptosis of myocardial cells in** **acute myocardial infarction: a study in vitro**

Xuemei Liu^1#^, Zulikaier Tuerxun^3#^, Yumaierjiang Balati^3^, Pengfei Gong^2^, Ze Zhang^3^, Zhen Bao^2^, Yuchun Yang^2^, Pengyi He^3*^, Muhuyati^2*^

1. Department of Respiratory Medicine, The First Affiliated Hospital of Xinjiang Medical University, Urumqi 830054, China

2. Department of Integrated Cardiology, The First Affiliated Hospital of Xinjiang Medical University, Urumqi 830054, China

3. The Second Department of Coronary Heart Disease, the First Affiliated Hospital of Xinjiang Medical University, Urumqi 830054, China

#These authors contributed equally to this work

*Corresponding authors:

Pengyi He

Email: [813058823@qq.com](mailto:813058823@qq.com), The First Affiliated Hospital of Xinjiang Medical University, No. 137, Liyushan South Road, Xinshi District, Urumqi 830054, China.

Muhuyati

Email: [mhyt567@163.com](mailto:mhyt567@163.com), The First Affiliated Hospital of Xinjiang Medical University, No. 137, Liyushan South Road, New urban, Urumqi 830054, China.

**Table S1 Dilution ratio of antibodies**

| First antibody | Dilution ratio | Second antibody | Dilution  ratio |
| --- | --- | --- | --- |
| β-actin | 1:1000 | Goat anti-mouse IgG H&L (HRP) | 1:15000 |
| BAX | 1:400 |  | 1:5000 |
| CHOP | 1:800 |  | 1:5000 |
| eIF2α | 1:800 | Goat anti-rabbit IgG H&L (HRP) | 1:5000 |
| ATF4 | 1:800 |  | 1:5000 |
| GRP78 | 1:800 |  | 1:5000 |
| BCL-2 | 1:400 |  | 1:5000 |

**Table S2 OD values of HL-1 cell growth**

|  | Well 1 | Well 2 | Well 3 | Well 4 | Well 5 | Mean | Standard deviation |
| --- | --- | --- | --- | --- | --- | --- | --- |
| Day1 | 0.242 | 0.326 | 0.324 | 0.218 | 0.287 | 0.279 | 0.048 |
| Day2 | 0.377 | 0.478 | 0.416 | 0.345 | 0.400 | 0.403 | 0.050 |
| Day3 | 0.634 | 0.636 | 0.637 | 0.562 | 0.572 | 0.608 | 0.038 |
| Day4 | 0.983 | 1.022 | 0.965 | 1.024 | 0.945 | 0.988 | 0.035 |
| Day5 | 1.130 | 1.136 | 1.173 | 1.138 | 1.149 | 1.145 | 0.017 |
| Day6 | 1.298 | 1.279 | 1.233 | 1.317 | 1.316 | 1.289 | 0.035 |
| Day7 | 1.302 | 1.293 | 1.371 | 1.297 | 1.306 | 1.314 | 0.032 |

**Table S3 Proliferation test results of cardiomyocytes during different hypoxia**

**periods (**$\bar{\boldsymbol{\chi}}\boldsymbol{\pm s}$**，*n*=5)**

| Group | OD value | | | |
| --- | --- | --- | --- | --- |
|  | Hypoxia 3h | Hypoxia 6h | Hypoxia 12h | Hypoxia 24h |
| Control | 0.249±0.027 | 0.492±0.027 | 0.669±0.054 | 0.891±0.038 |
| Hypoxia | 0.237±0.025 | 0.406±0.022^△^ | 0.430±0.032^△^ | 0.266±0.032^△^ |

Note: △ means compare with the control，*p*<0.05。

**Table S4 Effects of different hypoxia time on LDH activity of HL-1 cardiomyocytes (**$\bar{\boldsymbol{\chi}}\boldsymbol{\pm s}$**，*n*=3)**

| Group | LDH (U/L) | | | |
| --- | --- | --- | --- | --- |
|  | Hypoxia 3h | Hypoxia 6h | Hypoxia 12h | Hypoxia 24h |
| Control | 78.302±11.438 | 76.101±12.843 | 84.277±18.495 | 86.163±10.729 |
| Hypoxia | 82.704±8.024 | 144.025±15.338^△^ | 182.390±36.542^△^ | 262.264±16.583^△^ |

Note: △ means compare with the control，*p*<0.05。

**Table S5 Expression analysis of POSTN in cardiomyocytes infected**

**with GV492-POSTN-WT (**$\bar{\boldsymbol{\chi}}\boldsymbol{\pm s}$**，*n*=3)**

| Group | POSTN |
| --- | --- |
| Control | 1.021±0.256 |
| GV492-NC | 0.864±0.342 |
| GV492-POSTN-WT | 69.188±15.162^△▲^ |

Note: △ means compare with the control，*p*<0.05；▲ means compare with GV492-NC，*p*<0.05

**Table S6 Expression analysis of POSTN alternative splicing after GV492-POSTN-MUT infection of cardiomyocytes (**$\bar{\boldsymbol{\chi}}\boldsymbol{\pm s}$**，*n*=3)**

| Group | POSTN-full/β-actin | POSTN-short/β-actin |
| --- | --- | --- |
| Control | 0.711±0.009 | 2.437±0.405 |
| GV492-NC | 0.611±0.203 | 2.287±0.139 |
| GV492-POSTN-MUT | 1.284±0.152^▲^ | 2.464±1.566 |

Note：POSTN-full is the alternative splicing of POSTN，POSTN-short is the reference transcript，GV492-NC is the negative control lentivirus, GV492-POSTN-MUT is the POSTN mutant overexpressed lentivirus, △ means compare with the control，*p*<0.05；▲ means compare with GV492-NC，*p*<0.05.

**Table S7 Effects of GV492-POSTN-WT and GV492-POSTN-MUT on proliferation of cardiomyocytes (**$\bar{\boldsymbol{\chi}}\boldsymbol{\pm s}$**，*n*=5)**

| Group | OD value |
| --- | --- |
| Control | 0.825±0.031 |
| Hypoxia model | 0.423±0.046^△^ |
| GV492-NC | 0.744±0.054^△▲^ |
| GV492-POSTN-WT | 0.821±0.023^▲▽^ |
| GV492-POSTN-MUT | 0.809±0.073^▲^ |
| Hypoxia model +GV492-NC | 0.378±0.030^△▽▼◇^ |
| Hypoxia model +GV492-POSTN-WT | 0.543±0.056^△▲▽▼◇◆^ |
| Hypoxia model +GV492-POSTN-MUT | 0.480±0.068^△▽▼◇◆^ |

Note: △ means compare with the control, *p*<0.05; ▲ means compare with the hypoxia model，*p*<0.05; ▽ means compare with GV492-NC, *p*<0.05; ▼ means compare with GV492-POSTN-WT, *p*<0.05; ◇means compare with GV492-POSTN-MUT, *p*<0.05; ◆means compare with hypoxia model+GV492-NC, *p*<0.05; ☆ means compare with hypoxia model+GV492-POSTN-WT, *p*<0.05.

**Table S8 Effects of GV492-POSTN-WT and GV492-POSTN-MUT on myocardial cell apoptosis (**$\bar{\boldsymbol{\chi}}\boldsymbol{\pm s}$**，*n*=3)**

| Group | Apoptosis rate (%) |
| --- | --- |
| Control | 4.873±0.717 |
| Hypoxia model | 17.883±0.896^△^ |
| GV492-NC | 5.737±0.156^▲^ |
| GV492-POSTN-WT | 3.697±0.397^△▲▽^ |
| GV492-POSTN-MUT | 3.653±0.264^△▲▽^ |
| Hypoxia model +GV492-NC | 19.210±0.348^△▲▽▼◇^ |
| Hypoxia model +GV492-POSTN-WT | 12.050±0.573^△▲▽▼◇◆^ |
| Hypoxia model +GV492-POSTN-MUT | 14.410±0.631^△▲▽▼◇◆☆^ |

Note: △ means compare with the control, *p*<0.05; ▲ means compare with the hypoxia model，*p*<0.05; ▽ means compare with GV492-NC, *p*<0.05; ▼ means compare with GV492-POSTN-WT, *p*<0.05; ◇means compare with GV492-POSTN-MUT, *p*<0.05; ◆means compare with hypoxia model+GV492-NC, *p*<0.05; ☆ means compare with hypoxia model+GV492-POSTN-WT, *p*<0.05.

**Table S9 Expression analysis of apoptosis related protein eIF2α, ATF4 and CHOP in cardiomyocytes under different interventions (**$\bar{\boldsymbol{\chi}}\boldsymbol{\pm s}$**，*n*=3)**

| Group | **eIF2α** | **ATF4** | **CHOP** |
| --- | --- | --- | --- |
| Control | 0.409±0.013 | 0.474±0.063 | 0.358±0.082 |
| Hypoxia model | 0.717±0.062^△^ | 0.866±0.097^△^ | 0.758±0.083^△^ |
| GV492-NC | 0.441±0.051^▲^ | 0.466±0.058^▲^ | 0.380±0.124^▲^ |
| GV492-POSTN-WT | 0.272±0.051^△▲▽^ | 0.346±0.054^△▲▽^ | 0.229±0.080^▲▽^ |
| GV492-POSTN-MUT | 0.282±0.034^△▲▽^ | 0.378±0.049^▲^ | 0.237±0.059^▲▽^ |
| Hypoxia model +GV492-NC | 0.721±0.065^△▽▼◇^ | 0.866±0.089^△▽▼◇^ | 0.809±0.019^△▽▼◇^ |
| Hypoxia model +GV492-POSTN-WT | 0.532±0.038^△▲▽▼◇◆^ | 0.704±0.039^△▲▽▼◇◆^ | 0.561±0.089^△▲▽▼◇◆^ |
| Hypoxia model +GV492-POSTN-MUT | 0.495±0.064^▲▼◇◆^ | 0.660±0.027^△▲▽▼◇◆^ | 0.587±0.031^△▲▽▼◇◆^ |

Note: △ means compare with the control, *p*<0.05; ▲ means compare with the hypoxia model，*p*<0.05; ▽ means compare with GV492-NC, *p*<0.05; ▼ means compare with GV492-POSTN-WT, *p*<0.05; ◇means compare with GV492-POSTN-MUT, *p*<0.05; ◆means compare with hypoxia model+GV492-NC, *p*<0.05.

**Table S10 Expression analysis of apoptosis related protein GRP78, BCL-2 and BAX in cardiomyocytes under different interventions (**$\bar{\boldsymbol{\chi}}\boldsymbol{\pm s}$**，*n*=3)**

| Group | **GRP78** | **BCL-2** | **BAX** |
| --- | --- | --- | --- |
| Control | 0.384±0.035 | 0.585±0.025 | 0.598±0.047 |
| Hypoxia model | 0.616±0.017^△^ | 0.476±0.043^△^ | 0.886±0.089^△^ |
| GV492-NC | 0.392±0.014^▲^ | 0.611±0.047^▲^ | 0.606±0.032^▲^ |
| GV492-POSTN-WT | 0.286±0.019^△▲▽^ | 0.730±0.053^△▲▽^ | 0.507±0.046^▲▽^ |
| GV492-POSTN-MUT | 0.310±0.035^△▲▽^ | 0.713±0.026^△▲▽^ | 0.519±0.045^▲^ |
| Hypoxia model +GV492-NC | 0.624±0.025^△▽▼◇^ | 0.472±0.027^△▽▼◇^ | 0.904±0.037^△▽▼◇^ |
| Hypoxia model +GV492-POSTN-WT | 0.521±0.077^△▲▽▼◇◆^ | 0.563±0.029^▲▼◇◆^ | 0.688±0.034^▲▼◇◆^ |
| Hypoxia model +GV492-POSTN-MUT | 0.461±0.044^△▲▽▼◇◆^ | 0.549±0.058^▲▼◇◆^ | 0.676±0.076^▲▼◇◆^ |

Note: △ means compare with the control, *p*<0.05; ▲ means compare with the hypoxia model，*p*<0.05; ▽ means compare with GV492-NC, *p*<0.05; ▼ means compare with GV492-POSTN-WT, *p*<0.05; ◇means compare with GV492-POSTN-MUT, *p*<0.05; ◆means compare with hypoxia model+GV492-NC, *p*<0.05.


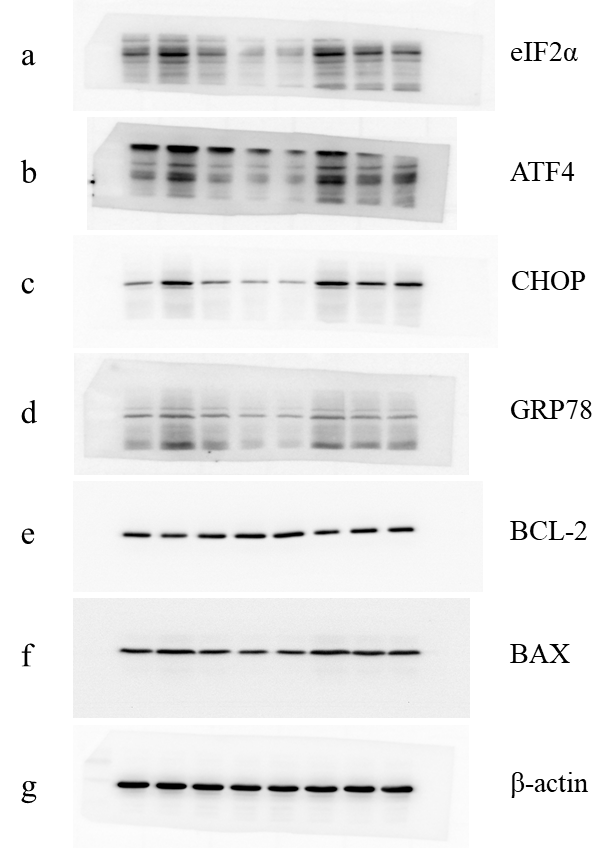


**Figure S1 Full length bands of apoptosis-related proteins detected by Western Blot**

The groups represented by the bands from left to right are: Control, Model, GV492-NC, GV492-WT, GV492-MUT, Model+GV492-NC, Model+GV492-WT, Model+GV492-MUT, and the “model” is a shorthand for “ischemic hypoxic myocardial cell model”.
